# Human interest meets biodiversity hotspots: A new systematic approach for urban ecosystem conservation

**DOI:** 10.1371/journal.pone.0172670

**Published:** 2017-02-24

**Authors:** Minoru Kasada, Misako Matsuba, Tadashi Miyashita

**Affiliations:** 1 Department of General Systems Studies, Graduate School of Arts and Sciences, The University of Tokyo, Meguro, Tokyo Japan; 2 Project Team for Analyses of Changes in East Japan Marine Ecosystems Japan Agency for Marine-Earth Science and Technology, Yokosuka, Kanagawa, Japan; 3 Department of Ecosystem Studies, Graduate School of Agricultural and Life Sciences, The University of Tokyo, Bunkyoku, Tokyo, Japan; Cary Institute of Ecosystem Studies, UNITED STATES

## Abstract

Creating a win-win relationship between biodiversity and human well-being is one of the major current challenges for environmental policy. One way to approach this challenge is to identify sites with both high biodiversity and high human interest in urban areas. Here, we propose a new systematic approach to identify such sites by using land prices and biodiversity indexes for butterflies and birds from a nationwide perspective. As a result, we found sites that are valuable to humans and to other organisms, including national red-list species, and they are located in sites with cultural heritages and near seaside. By referencing the habitat features and landscape characteristics of these sites, we can establish high quality environments that provide a benefit to both humans and biodiversity in urban landscapes.

## Introduction

It is estimated that 66% of the world's human population will live in urban areas by 2050 [1]. In this context, promoting biodiversity in the urban ecosystem is thought to be a viable way to decelerate the rapid loss of global biodiversity as intact areas of natural habitats are lost [2]. In addition, recent studies have shown that there are increased psychological benefits associated with access to urban green space [3–5] and wildlife [6–8]. Moreover, urban green spaces are valuable in providing the experience of nature [9] to people living in cities, which may help prevent the disengagement of people with nature ('extinction of experience') [10] and maintain motivation for biodiversity conservation. However, recent urbanization generally leads to biological uniformity and people in urban areas encounter only common, widespread, and alien species [11, 12], which have a lower value for biological conservation. It is therefore, a benefit for urban green spaces to have populations of a variety of native species to facilitate a sense of nature for human inhabitants and to improve biodiversity [13–17]. In this context, we need to conserve native biodiversity in areas with a large human population density or frequent visitation because such areas provide high opportunities for urban people to access biodiversity. Thus, places with both high biodiversity and high human interest appear to be valuable for keeping human-biodiversity relationships in urban settings, and such places should be given a high priority for conservation.

To search such places in an objective way, we developed a new systematic tool by using land prices and an index of biodiversity. We used land price as a measure of human interest, because it represents an integrated measure of human interest. Population density could be another measure of human interest, but there are some urban areas where many people may frequently use without actually residing there, such as business and factory districts. Because such areas have lower resident population densities, land price is considered better suited for our research aim, rather than human population density. Areas with high land price and high biodiversity (hereafter, high-high sites) would have high ecosystem value [18–20], since such areas could act as important meeting points of biodiversity and people.

In this paper, we identified high-high sites across Japan by searching for local maximal values of (biodiversity) x (land price), using nation-wide datasets for butterflies, birds, and land prices. The reason for choosing butterflies and birds is that nation-wide data of almost all species are available in Japan and they are the representative taxonomic groups often used for biodiversity index in earlier studies [21]. The rationale for searching the local maximum high-high sites is that the spatial scale is compatible with that of local government, which is useful for the implementation of local policies and regional biodiversity strategy. Based on these results, we discuss why these sites became high-high sites, and provide suggestions for the conservation and management of urban ecosystems.

## Methods

### Model

To detect sites with both high land price and high biodiversity (high-high sites), we developed a new index *V*_*h-h*_, which is defined as

$$V_{h-h}=\alpha \;\log\;l+\beta \;\log\;b$$

where *l* is land price, *b* is an index for species richness, and α and β are weighting parameters. These parameters can be changed for a purpose, that is, β is set large when human value is prioritized, while α is set large when biodiversity is prioritized. Here we set α = β = 1 to make no such bias.

**Fig 1:**
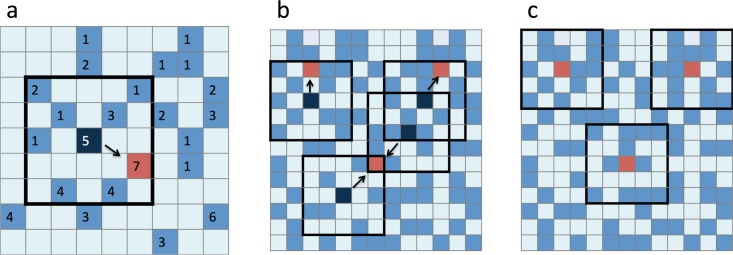
Simulation algorithm for estimating local maximal points of *V*_*h-h*_. (a) Search the new point (red site) with the highest *V*_*h-h*_ in a given range around a focal point (dark blue site); (b) obtain a set of points with highest values (red sites); and (c) no new set of points are found.

We conducted the local search algorithm and found maximal values on regional scale across the country. The initial search range was determined so that the number of selected sites in Japan resulted in a total of 10 to 20, which roughly corresponds to the number of major regions in Japan. Increasing the search range decreased the number of the selected sites (S1 Fig). The actual search range we chose differed between butterflies and birds, depending on the number of data points available, and the details are described in the next section (see Case studies). To make the selection of local maximal values, we conducted the following simulation algorithm:

1. Calculate *V*_*h-h*_ for the all data points on a nation-wide scale.
2. Search the new point with the highest *V*_*h-h*_ in the search range around a point (Fig 1A).
3. Apply step 2 for all the data points and obtain a new set of those with the highest *V*_*h-h*_ values (Fig 1B).
4. Repeat step 3 for the set until the selected set of points is no longer changed (Fig 1C).

After this simulation, we referenced the details of land use type of the selected sites.

### Case studies: Selection for high-high sites

We applied the above method to nation-wide distribution data of butterflies and birds in Japan. The data of land prices in 2015 were obtained from the National Land Numerical Information download service at the Japanese Ministry of Land, Infrastructure, Transport and Tourism [22]. For land prices in the 1 km × 1 km grid cells, only a small portion of the whole area of Japan was available (4.3%), but more than 35% of the total urban areas (residential areas >20%) were available. Distribution data for butterflies were obtained from the fourth and fifth National Surveys on the Natural Environment from 1988 to 1998, which were the most recent and extensively surveyed data available in Japan at the time of our study. We used distribution data for 66 butterfly species that had the resolution of 1 km × 1 km grid cells, by implementing the MaxEnt model (see S1 Text). For the additional 11 species whose distributions were highly restricted, we used raw data of their distributions instead of using models. As a result, we used the data for 77 butterfly species. We defined an index of butterfly species richness weighted by the distribution area for each species as:

$$\sum_{k}\frac{p_{kj}}{\sum_{l}p_{kl}}$$

where *p*_*ij*_ represents whether species *i* at point *j* exists (equal to 1) or not (equal to 0).

**Fig 2:**
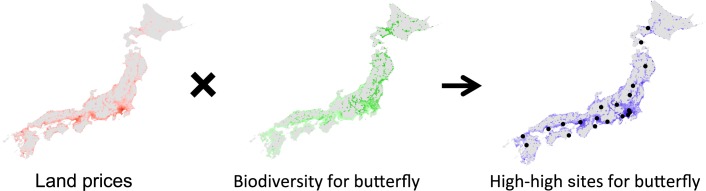
Distribution of land prices, species richness index of butterflies, and *V*_*h-h*_ value in Japan. Black circles in the rightmost panel indicate selected sites (10 km × 10 km grid cells) with a local maximum *V*_*h-h*._ Darker colors indicate higher values on all panels. Note that for the species richness index and *V*_*h-h*_, only sites where land price data were available (gray color means data not available).

After the values of land prices and species richness were normalized so that the sum of the all data equals to unity, *V*_*h-h*_ was calculated across the 4 main-islands of Japan (Hokkaido, Honsyu, Shikoku, and Kyusyu) with 1 km × 1 km grid cells (Fig 2). We obtained 15,395 cells with data values and calculated *V*_*h-h*_ for each cell. When a cell held more than two land prices, the average price was used. Here, we set the search range to more than 600 cells (mean: 119.2 km square). Since cells with data values are not distributed uniformly in Japan, the search ranges were from 600 to 691 cells.

We then tried to find green spaces that were supposed to be inhabited by birds and butterflies within the selected sites by using a map and aerial photos (Google map API). We checked the photos and maps of nine cells including eight neighboring cells around the selected sites to find green spaces that are likely to have higher species richness (see S2, S3, S4 and S5 Figs).

Finally, we calculated the mean number of rare species of butterflies in the selected cells. Here, rare species is defined as species belonging to Near Threatened or higher ranks in the of Japan. All of the sites used for the analysis (15,395 cells) included 21 rare butterfly species.

We also analyzed bird data in Japan using our land and biodiversity index. The land price data were the same as in the butterfly case and we used the distribution data of 172 (MaxEnt model: 97, raw data: 75) terrestrial breeding bird species in Japan [23], which had the resolution of 10 km × 10 km grid cells. The resolution for the birds is coarser than that for the butterflies because the finer scale data are not open to public use. The nation-wide distribution of birds was estimated from the MaxEnt models based on the data of from the second National Survey on the Natural environment 1978 and from surveys spanning from 1997 to 2002 (for details see [23]). As a result, we obtained 1662 cells with data values and calculated *V*_*h-h*_ for each cell. The search range set included more than 100 cells (mean: 239.6 km square) with data values ranging from 100 to 134, to which the simulation algorithm was applied. As in the case for the butterflies, this search range was determined such that the number of selected sites was from 10 to 20. We checked details of land use type in the selected cells and their neighboring eight cells by using a map and aerial photos (Google map API), and then calculated the mean number of rare species in the selected cells. All of the cells used for the analysis included 45 rare bird species in total.

## Results

### Case studies: Selection for high-high sites

Local maximal values of *V*_*h-h*_ for butterflies in different 20 sites were selected as the high-high sites (Fig 2). There were three different land usage types within these selected sites; eleven sites were urban areas that included large green spaces (S2 Fig), three were small towns surrounded by natural green spaces (S3 Fig), and the remaining six sites were complex mixtures of green spaces and residential sections (S4 Fig). It was notable that more than half (55%, 11/20) of the green spaces selected were a Japanese "Shiro" (castle) or ruins of a "Shiro”, and this proportion was much larger than that from the other sites (15%). The mean number of rare butterfly species in these selected high-high sites was 1.75 ± 0.36 (S.E.), compared to 0.78 ± 0.01 (S.E.) in the other sites.

**Fig 3:**
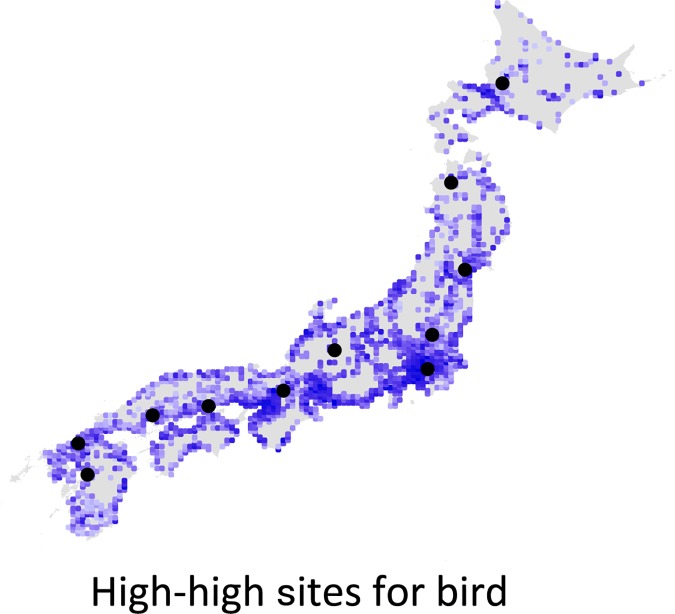
Distribution of *V*_*h-h*_ for birds. Black circles indicate selected sites (1 km × 1 km grid cells) with locally maximum *V*_*h-h*._ Darker colors indicate higher values. Note that for the species richness index and *V*_*h-h*_, only sites where land price data are available (gray color means data not available).

For birds, eleven sites were selected that had local maximal values of *V*_*h-h*_ (Fig 3). The mean number of rare bird species in these sites was 4.55 ± 0.67 (S.E.), compared to a mean of 3.02 ± 0.04 (S.E.) species in the other sites. Seven sites were located in major cities in a region along a seaside and lakeside (S5 Fig). Among these sites, five sites were overlapped with the high-high sites for butterflies.

## Discussion

Although green space in and around urban areas has been considered important for providing ecosystem services and for providing animal habitats [2, 24–26], they have not been well assessed in consideration of an integrative estimate of biodiversity and human value. Most conservation efforts have focused on maintaining intact natural habitats that are generally not located in urban areas. Here, we found sites that have high diversity of butterfly or bird species relative to their urbanization degree by using a novel index of *V*_*h-h*_. We found that the selected sites include some Red List species of butterflies and birds, and the number of such species is higher than that found in the non-selected sites on a nation-wide scale. As butterflies and birds are usable as indicators for biodiversity in general and land price is good indicator of human value, our systematic approach is effective in finding local green spaces with high biodiversity in and around urban areas using a nation-wide perspective. Consequently, our approach could provide scientifically-based evidence for priority setting in urban planning and management policies, as well as in regional biodiversity strategy.

Interestingly, many of the high-high sites for butterflies included Japanese "Shiro" or ruins of "Shiro". These areas are regarded as cultural heritage areas rather than protected natural refuges. Another cultural heritage documented in this butterfly analysis is an "Ise" (a shrine). Since these cultural heritages are known to harbor higher plant species richness in comparison to their surrounding environments [27, 28], abundant host and nectar plants might have maintained rich butterfly fauna there. This indicates that protecting cultural heritage could lead to the protection of natural ecosystems as well, probably because the environment may not have been disturbed by human activities for the sake of preserving scenery and sacredness. Previous studies have shown a higher species richness in cultural heritage sites in urban areas, but they have some methodological problems as the sites compared were arbitrarily chosen at local or regional scales [29–31]. In contrast, we used systematic approach that was conducted in a nation-wide scale, allowing us to identify locally important sites from a nation-wide perspective. We suggest that it is necessary to maintain the environmental quality of cultural heritage sites in a whole landscape, even if cultural buildings are no longer presently used or maintained.

For birds, many of the high-high sites were located in large local cities along the seaside and estuaries that included prefectural capitals, while others were located in cities surrounded by natural green spaces. Because Red List bird species in Japan includes many wading or water birds, such sites may be specifically attractive to these species. This information can encourage local governments and NPO (Non-Profit Organization) conservation actions or policies that will maintain green space and seaside environments within city limits.

There are potential caveats with our study. First, butterfly and bird data are based on older surveys relative to land price data. However, the cultural heritage sites we identified as important (high-high) urban green spaces had existed for more than several hundred years, and urban sprawl has been diminished in these decades due to economic slowdown in Japan. Therefore, the major conclusion would not have changed substantially if we could have had obtained new biodiversity data. Second, there is uncertainty in the outputs of MaxEnt model, which might have influenced *V*_*h-h*_ values. However, as we used many species for the modeling (66 for butterflies, 97 for birds), estimation bias is likely to have been cancelled out.

There are also other important issues still to be addressed. First, we used only two taxonomic groups in this study and although they represent good indicators for biodiversity in urban areas, adding more groups would expand on the usefulness of our method. An analysis that uses more taxonomic groups would not only provide general information on space use by different species, but would benefit the decision making process at practical and policy levels [32]. Second, applying our systematic method to other nations or biomes is likely to discover other high-high site with different environmental features and land-cover types, which may be determined by regional species pools. Finally, we would like to emphasize that our new method is able to find win-win places objectively by searching local maximum *V*_*h-h*_ values, and if the searching range in the algorithm is decreased, we can find a greater number of local maxima across an entire nation. Such flexibility could help implement local environmental policies and regional biodiversity strategies at various spatial scales. We hope our study aids in establishing urban ecosystem management that can lead to the co-prosperity of humans and wildlife.

## Supporting information

S1 Fig Relationships between the search range and the number of the selected sites. (TIFF)

S2 Fig An aerial photograph of a typical urban area including large green spaces (Google map API). (TIFF)

S3 Fig An aerial photograph of typical small town area that is enclosed in a natural green space (Google map API). (TIFF)

S4 Fig An aerial photograph of a typical area with a complex mixture or edge of green spaces and residential sections (Google map API). (TIFF)

S5 Fig An aerial photograph of a typical area located in local cities along a seaside or lakeside (Google map API). (TIFF)

S1 Text Species distribution models. (DOCX)
