# Supplementary figures and images for: Human interest meets biodiversity hotspots: A new systematic approach for urban ecosystem conservation
Source: PLoS One. 2017 Feb 24;12(2):e0172670. doi: 10.1371/journal.pone.0172670 (PMC5325308; doi:10.1371/journal.pone.0172670)

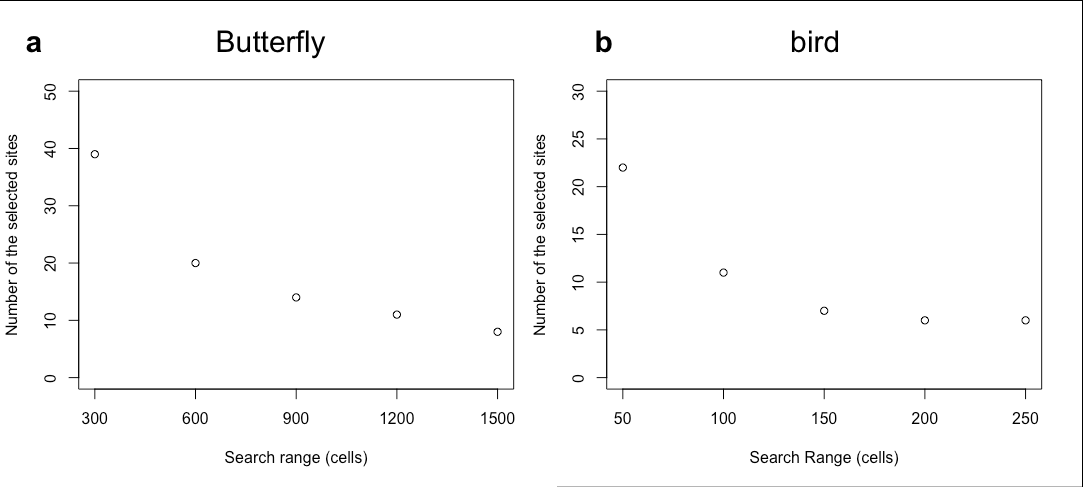

Supplement: S1 Fig — (TIFF) [file pone.0172670.s001.tiff]

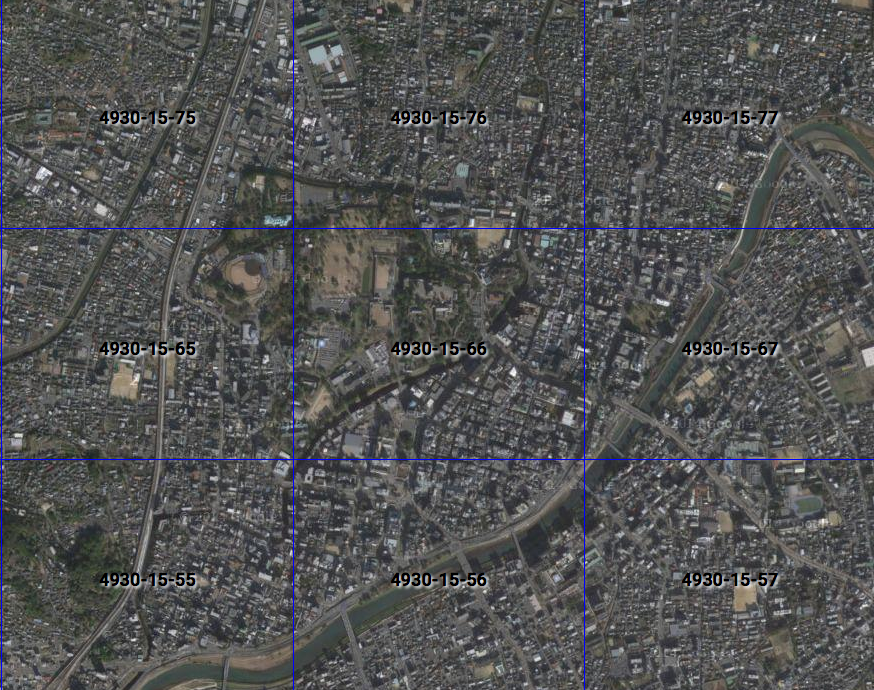

Supplement: S2 Fig — (TIFF) [file pone.0172670.s002.tiff]

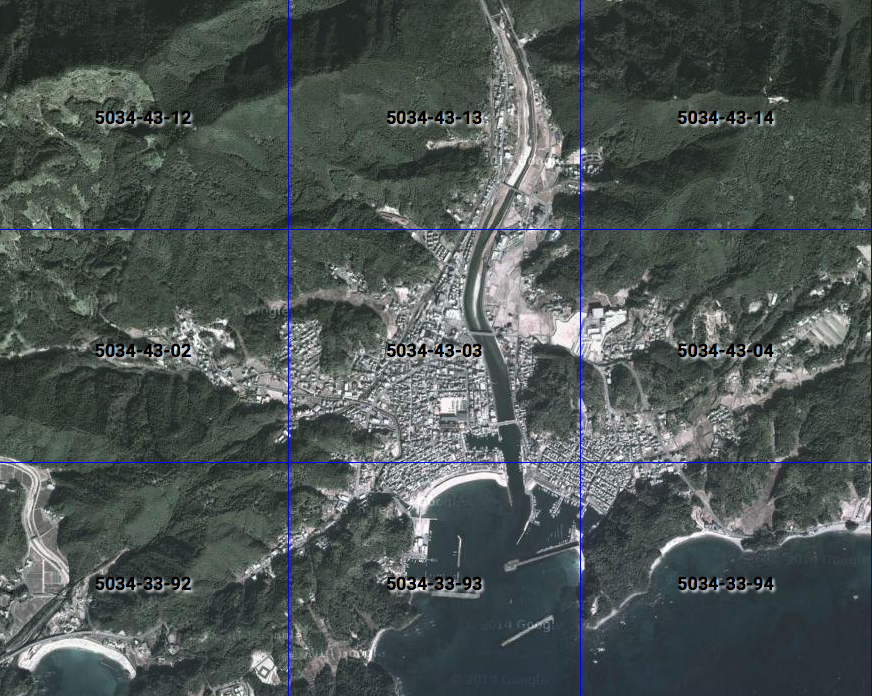

Supplement: S3 Fig — (TIFF) [file pone.0172670.s003.tiff]

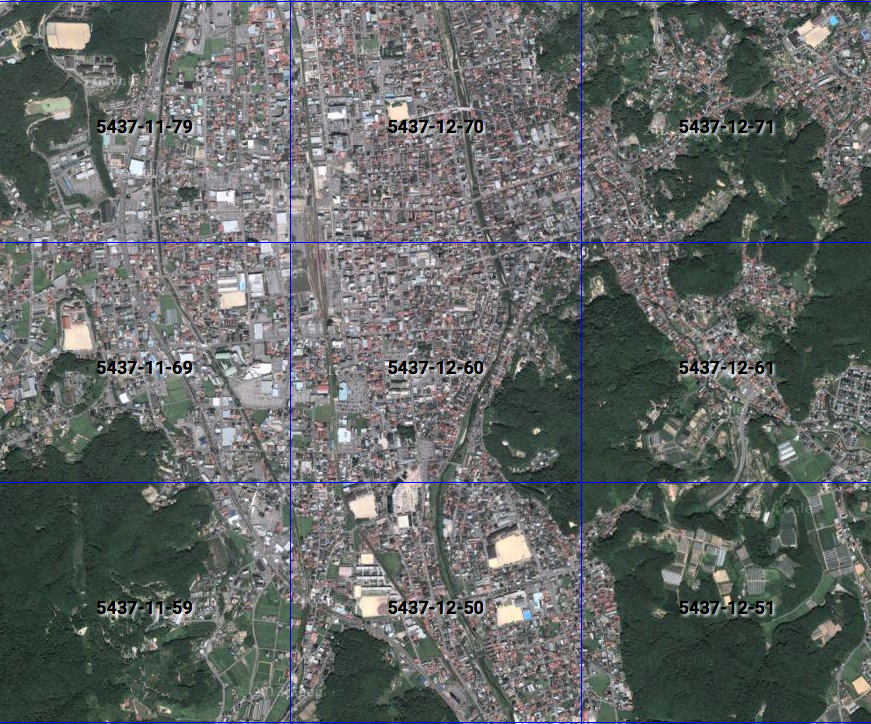

Supplement: S4 Fig — (TIFF) [file pone.0172670.s004.tiff]

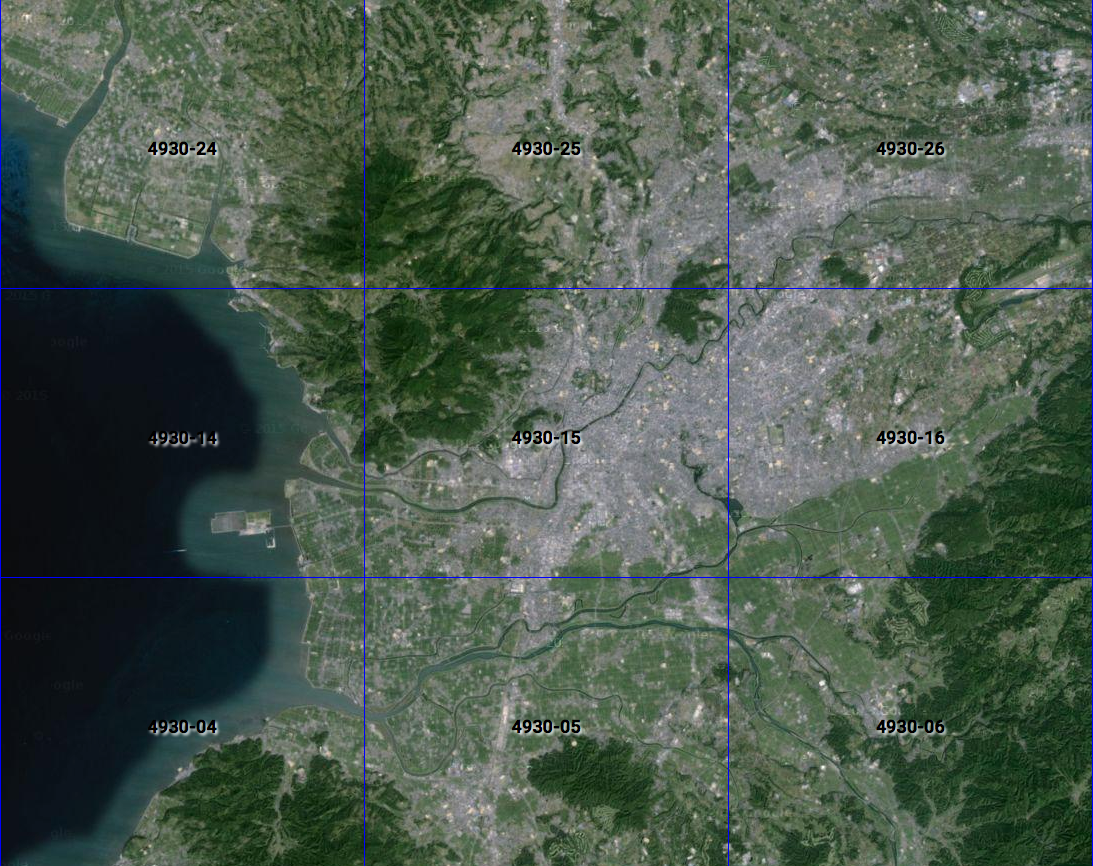

Supplement: S5 Fig — (TIFF) [file pone.0172670.s005.tiff]
