# Supplementary material for: Human interest meets biodiversity hotspots: A new systematic approach for urban ecosystem conservation
Source: PLoS One. 2017 Feb 24;12(2):e0172670. doi: 10.1371/journal.pone.0172670 (PMC5325308; doi:10.1371/journal.pone.0172670)
Supplement: S1 Text — (DOCX) [file pone.0172670.s006.docx]

**S1 text**

**Species Distribution Models**

We constructed species distribution models for each species with MaxEnt that provides high predictability using presence-only data, particularly with fewer occurrences. Raw distribution data for butterflies were obtained from the fourth and fifth National Surveys of the Natural Environment from 1988 to 1998, which are the most recent and extensively surveyed data available in Japan. We used 10 landscape variables (mean elevation; area proportions of broad-deciduous forests, broad-evergreen forests, conifer-evergreen forests, other forests, inland water including wetland, grasslands, agricultural fields, and paddy fields; and Simpson’s landscape diversity index) and two climate variables (annual mean temperature and annual precipitation) for the species distribution modeling. Mean elevation was collected from the Digital National Land Information project of the Japanese Ministry of Land, Infrastructure, Transport and Tourism. Eight land use variables other than Simpson’s diversity index were calculated from the data of Ogawa et al. (2013)[1] and Akasaka et al. (2014)[2], which were based on the fifth national vegetation map produced by the Japanese Ministry of the Environment from 1994 to 1998. Simpson’s diversity index was calculated as 1- Σ*p_i_*^2^, where *p_i_* is the area proportions of land use type *i*, composed of broad-deciduous forests, broad-evergreen forests, conifer-evergreen forests, other forests, grasslands, agricultural fields, or paddy fields. Climate data were derived from Mesh Climate Value 2000, provided by the Meteorological Agency of Japan. All environmental data were created in a 1 km x 1 km resolution. We checked the multicollinearity among the variables using the variance inflation factor (VIF), and found no serious multicollinearity (VIFs < 10).

In conducting MaxEnt, we first estimated the probability of habitat suitability (continuous values scaled 0 to 1) for each species, and then transformed them into binary values (zero (unsuitable) or 1 (suitable)) with an appropriate threshold to make distribution maps. We used different thresholds for the different species that were determined by expert judgment, whereby tuning the estimated distribution to atlas information [3]; minimum training presence logistic threshold for common species, 0.5 for three species (a relatively higher threshold value to avoid overestimation), and maximum training presence logistic threshold for the others. Also, we restricted the prediction area for 17 species that have clear biogeographic ranges, which were determined by surveying existing literature and expert opinion (the restricted areas were mainly defined as islands, such as Hokkaido, Honshu, Kyushu, Shikoku, and a chain of mountains which could function as a barrier for butterflies).

Estimations for species distribution were conducted using the following settings in the MaxEnt software; the default “auto features” option, logistic output, and the maximum number of iteration (500). We used a target group background method for extracting pseudo-absence data. Phillip et al. (2009)[4] suggested that this method improves accuracies of inferences because it uses background data from occurrences of similar species groups with similar sampling biases. For each run, we randomly divided the occurrence data into 70% for calibration and 30% for validation of the models. To evaluate the model performance, we calculated the area under the curve (AUC) from the receiver operating characteristic (ROC) plots. To account for model uncertainty, we used the averaged values for the inferences over 10 replicates of each run. We assumed the model with AUC > 0.6 had acceptable model performance, which resulted in model construction for 153 species. The species with AUC < 0.6 were very common throughout Japan and we excluded these species from the following analyses. Besides the AUC evaluation, we adopted expert opinion to judge whether the models with AUC > 0.6 were really valid. We did this because evaluation with AUC could lead to an overestimation when using a presence-only data. As a result, models of 66 species turned out to be valid. For the additional 11 species whose distributions were highly restricted, we used raw data of their distributions instead of using models.

**Refarences**

1. Ogawa M, Takenaka A, Kadoya T, Ishihama F, Yamano H, Akasaka M. Land-use classification and mapping at a whole scale of Japan based on a national vegetation map. Japanese J Conserv Ecol. 2013; 18: 69–76 (in Japanese with English summary).

2. Akasaka M, Takenaka A, Ishihama F, Kadoya T, Ogawa M, Osawa T, Yamakita, T, et al. Development of a national land-use/cover dataset to estimate biodiversity and ecosystem services. In: Nakano S, Yahara T, Nakashizuka T, editors. The biodiversity observation network in the Asia-Pacific region: Integrative observations and assessments of Asian biodiversity. Tokyo: Springer; 2014. p. 209–229.

3. Japan Butterfly Conservation Society, editors. Field guide to the butterflies of Japan. 2nd ed. Tokyo: Seibundo-Shinkosha; 2012. Japanese

4. Phillips SJ, Dudik M, Elith J, Graham CH, Lehmann A, Leathwick J, et al. Sample selection bias and presence-only distribution models: implications for back-ground and pseudo-absence data. Ecol. Appl. 2009; 19: 181–197.
